# Crystal structure and Hirshfeld surface analysis of 1-carb­oxy-2-(3,4-di­hydroxy­phen­yl)ethan-1-aminium chloride 2-ammonio-3-(3,4-di­hydroxy­phen­yl)propano­ate: a new polymorph of l-dopa HCl and isotypic with its bromide counterpart

**DOI:** 10.1107/S2056989016016789

**Published:** 2016-10-25

**Authors:** Perumal Kathiravan, Thangavelu Balakrishnan, Perumal Venkatesan, Kandasamy Ramamurthi, María Judith Percino, Subbiah Thamotharan

**Affiliations:** a Crystal Growth Laboratory, PG and Research Department of Physics, Periyar EVR Government College (Autonomous), Tiruchirappalli 620 023, India; b Laboratorio de Polímeros, Centro de Química Instituto de Ciencias, Benemérita Universidad Autónoma de Puebla (BUAP), Complejo de Ciencias, ICUAP, Edif. 103H, 22 Sur y San Claudio, CP 72570 Puebla, Puebla, Mexico; c Crystal Growth and Thin Film Laboratory, Department of Physics and Nanotechnology, SRM University, Kattankulathur 603 203, India; d Biomolecular Crystallography Laboratory, Department of Bioinformatics, School of Chemical and Biotechnology, SASTRA University, Thanjavur 613 401, India

**Keywords:** crystal structure, l-dopa, cyclic N—H⋯Cl hydrogen bonds, Hirshfeld surfaces

## Abstract

The title mol­ecular salt, C_9_H_12_NO_4_^+^·Cl^−^·C_9_H_11_NO_4_, is isotypic with that of the bromide counterpart [Kathiravan *et al.* (2016 ▸). *Acta Cryst.* E**72**, 1544–1548]. The title salt is a second monoclinic polymorph of the l-dopa HCl structure reported earlier in the monoclinic space group *P*2_1_ [Jandacek & Earle (1971 ▸). *Acta Cryst.* B**27**, 841–845; Mostad & Rømming (1974 ▸). *Acta Chemica Scand.* B**28**, 1161–1168]. In the title compound, monoclinic space group *I*2, one of the dopa mol­ecules has a positive charge with a protonated α-amino group and the α-carb­oxy­lic acid group uncharged, while the second dopa mol­ecule has a neutral charge, the α-amino group is protonated and the α-carb­oxy­lic acid is deprotonated. In the previously reported form, a single dopa mol­ecule is observed in which the α-amino group is protonated and the α-carb­oxy­lic acid group is uncharged. The invariant and variations of various types of inter­molecular inter­actions present in these two forms of dopa HCl structures are discussed with the aid of two-dimensional fingerprint plots.

## Abstract 2

The crystal structure of new monoclininc polymorph of l-dopa HCl is reported, and hydrogen-bonding inter­actions are discussed.

## Chemical context   

The aromatic amino acid enzyme, tyrosine-3-hy­droxy­lase, catalyses the conversion of the amino acid l-tyrosine to l-dopa (l-3,4-di­hydroxy­phenyl­alanine). After successful conversion, the l-dopa mol­ecule acts as a precursor for neurotransmitter mol­ecules, such as dopamine, nor­epin­ephrine and epinephrine. The l-dopa mol­ecule is found to be an effective drug in the symptomatic treatment of Parkinson’s disease (Chan *et al.*, 2012 ▸). Polymorphism is very common amongst pharamaceutically important mol­ecules and is responsible for differences in many properties (Bernstein, 2002 ▸, 2011 ▸; Nangia, 2008 ▸; Guranda & Deeva, 2010 ▸). The first monoclinic form (I) [space group *P2_1_* and z′ = 1] of l-dopa HCl was reported in the 1970s (Jandacek & Earle, 1971 ▸; Mostad & Rømming, 1974 ▸). Herein, we report on the crystal and mol­ecular structure of a second monoclinic polymorph, form (II) (space group *I*2) of l-dopa HCl. The hydrogen-bonding patterns and the relative contributions of various inter­molecular inter­actions present in forms (I) and (II) are compared.
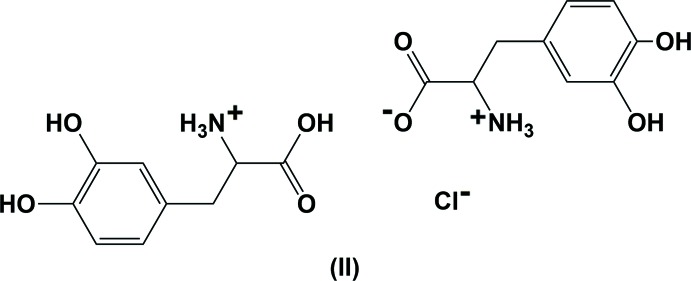


## Structural commentary   

**Figure 1:**
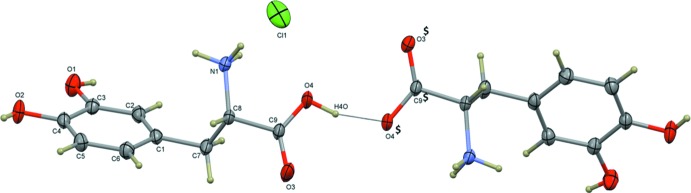
The mol­ecular structure of the title mol­ecular salt, (II), showing the atom labelling scheme [symmetry code: ($) −*x* + 3, *y*, −*z* + 1]. Displacement ellipsoids are drawn at the 50% probability level.

The asymmetric unit of the title compound, (II), is illustrated in Fig. 1 ▸. It consists of two dopa mol­ecules, and a Cl^−^ anion located on a twofold rotation axis. As observed in the isotypic l-dopa HBr mol­ecular salt (III) (Kathiravan *et al.*, 2016 ▸), one of the dopa mol­ecules is in the zwitterionic form and the other in the cationic form. In the cationic dopa mol­ecule, the α-amino group is protonated and carries a positive charge and the hydrogen atom (H4*O*) of the α-carb­oxy­lic acid group is located in a general position and was refined with 50% occupancy.

**Figure 2:**
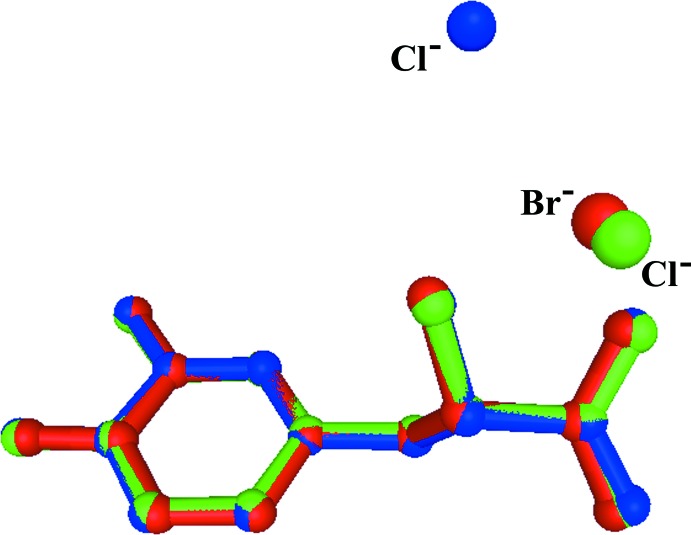
Structural superimposition of cationic dopa mol­ecules in (II) (red), bromide counterpart (green) and form (I) l-dopa HCl (blue).

The crystal structures of l-dopa (Mostad *et al.*, 1971 ▸), its hydro­chloride form (I) (Jandacek & Earle, 1971 ▸; Mostad & Rømming, 1974), the hydro­bromide form (III) (Kathiravan *et al.*, 2016 ▸) and the dihydrate form (André & Duarte, 2014 ▸), have been reported. The dihydrate form of dopa crystallizes in the ortho­rhom­bic space group *P*2_1_2_1_2_1_ with a single dopa mol­ecule in its zwitterionic form. The free dopa mol­ecule and its hydro­chloride form (I) crystallized in the monoclinic space group *P*2_1_. In the l-dopa structure, the dopa mol­ecule is in the zwitterionic form, while in the latter the α-amino group is proton­ated and the α-carb­oxy­lic acid is neutral. As mentioned earlier (Kathiravan *et al.*, 2016 ▸), the deposited coordinates of the l-dopa HCl structure belong to the *R* configuration. Therefore, the l-dopa HCl structure was inverted and the inverted model used for superposition. As shown in Fig. 2 ▸, one of the dopa mol­ecules of the title mol­ecular salt (II) is superimposed with the inverted model of l-dopa HCl (I) and one of the dopa mol­ecules of the isotypic Br compound (III). The r.m.s. deviation of the former pair is 0.105 Å while for the latter pair it is calculated to be 0.094 Å.

## Supra­molecular features   

**Figure 3:**
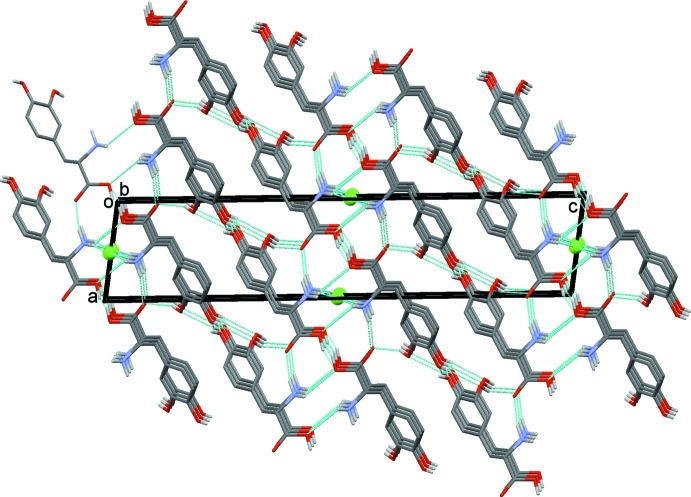
Crystal packing of the title mol­ecular salt, (II), viewed along the *b* axis. The hydrogen bonds are shown as dashed lines (see Table 1 ▸), and C-bound H atoms have been omitted for clarity.

**Figure 4:**
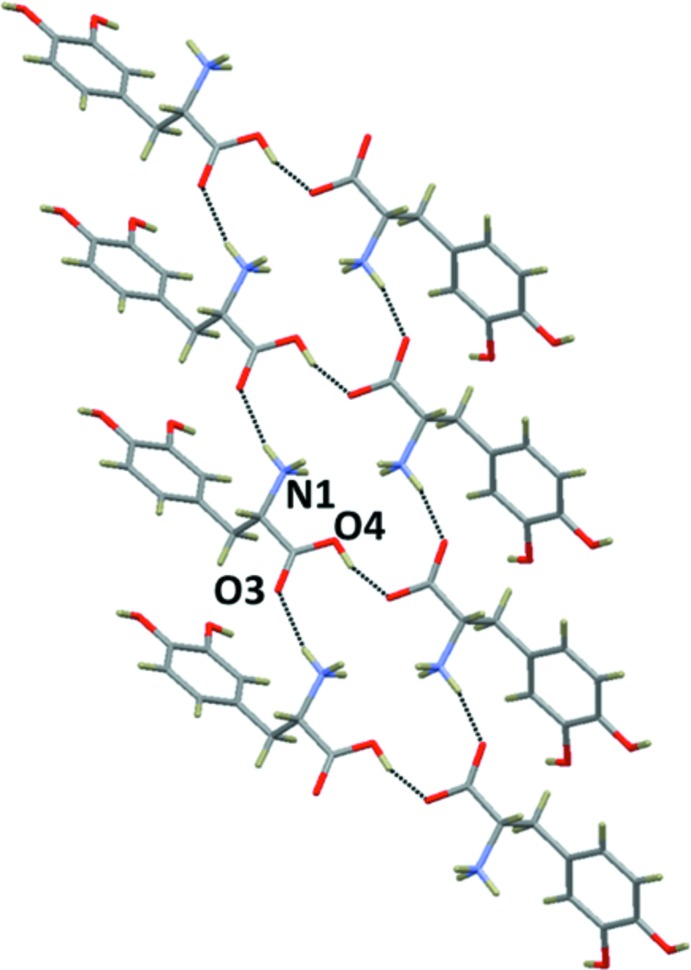
Part of the crystal structure of (II) showing the 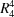
(18) motifs formed through N—H⋯O and O—H⋯O hydrogen bonds (see Table 1 ▸).

The crystal structure of the title mol­ecular salt (II) displays a network of inter­molecular N—H⋯Cl, N—H⋯O and O—H⋯O hydrogen bonds (Table 1 ▸), producing a three-dimensional framework (Fig. 3 ▸). It is of inter­est to note that the N—H⋯O and O—H⋯O hydrogen-bonding geometries in the title compound are slightly different when compared to its isotypic bromide counterpart (III) (Kathiravan *et al.*, 2016 ▸). A short inter­molecular O—H⋯O hydrogen bond links the carb­oxy­lic acid group of a dopa mol­ecule with the carboxyl­ate group of an adjacent dopa mol­ecule. This inter­action produces dopa dimers that are arranged as ribbons propagating along the *b* axis (Fig. 3 ▸). As observed in the bromide counterpart (III), the protonated amino group acts as a threefold donor for three inter­molecular hydrogen bonds, two of them with Cl^−^ anions and one with the carbonyl oxygen atom, O3, of the dopa acid group. One of the characteristic features observed in many amino acid–carb­oxy­lic acid/metal complexes (Sharma *et al.*, 2006 ▸; Selvaraj *et al.*, 2007 ▸; Balakrishnan, Ramamurthi & Thamotharan *et al.*, 2013 ▸; Balakrishnan, Ramamurthi, Jeyakanthan *et al.*, 2013 ▸; Sathiskumar *et al.*, 2015*a* ▸,*b* ▸,*c* ▸; Revathi *et al.*, 2015 ▸) is that the amino acid mol­ecules aggregate in head-to-tail sequences of the type ⋯NH_3_^+^—CH*R*—COO^−^⋯NH_3_^+^—CH*R*—COO^−^⋯ in which α-amino and α-carboxyl­ate groups are brought into periodic hydrogen-bonded proximity in a peptide-like arrangements. Similar arrangements (as layers) are observed in the title compound, in which α-amino (atom N1) and α-carboxyl­ate (atom O3) groups inter­act *via* an N—H⋯O hydrogen bond. Adjacent layers are inter­connected by strong O—H⋯O hydrogen bonds. The former N—H⋯O and the latter O—H⋯O inter­actions collectively form an 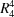
(18) ring motif (Fig. 4 ▸). Similar inter­actions are presented in dopa and the HCl form (I).

**Figure 5:**
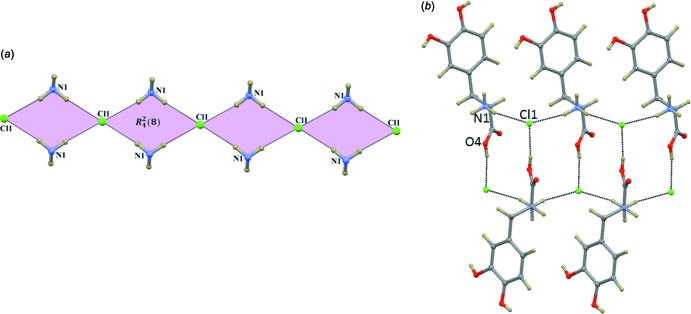
(*a*) Part of the crystal structure of (II) showing the 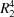
(8) motif formed by inter­molecular N—H⋯Cl hydrogen bonds (see Table 1 ▸), and (*b*) part of the crystal structure of form (I) dopa HCl showing the cyclic motif formed by N—H⋯Cl and O—H⋯Cl hydrogen bonds.

As shown in Table 1 ▸, the amino group (*via* H1*A* and H1*B*) of the dopa mol­ecule participates in N—H⋯Cl inter­actions with two different Cl^−^ anions. As observed in the bromide counterpart (III), these inter­actions inter­connect the cations and anions into a chain of cyclic motifs that enclose 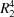
(8) rings and runs parallel to the *b* axis (Fig. 5 ▸ *a*). Forms (I) and (II) of the dopa HCl structures differ in the formation of cyclic motifs. In form (I), two N—H⋯Cl hydrogen bonds link the cations and anions into a chain. Adjacent chains are inter­connected through O—H⋯Cl inter­actions (carb­oxy­lic acid⋯Cl). Collectively, these inter­actions generate cyclic motifs (Fig. 5 ▸ *b*).

**Figure 6:**
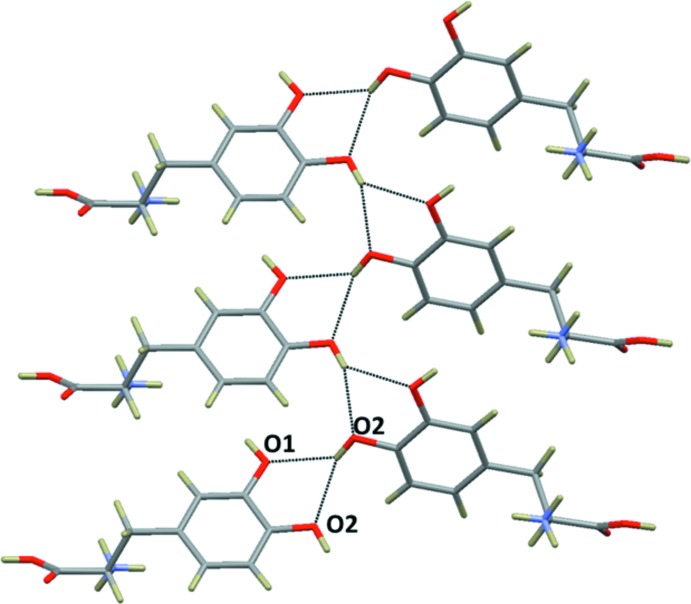
Adjacent dopa layers are inter­linked by side chain–side chain inter­actions in (II) through inter­molecular O—H⋯O hydrogen bonds (see Table 1 ▸).

The side-chain hy­droxy groups (O1—H1*O* and O2—H2*O*) of the dopa mol­ecules are involved in O—H⋯O hydrogen-bonding inter­actions, the former with the carbonyl oxygen atom (O3) and the latter in a bifurcated mode with two different hy­droxy (O1 and O2) oxygen atoms of adjacent dopa layers (Fig. 6 ▸). These inter­actions are invariant in the dopa structures reported earlier.

## Hirshfeld surface analysis   

**Figure 7:**
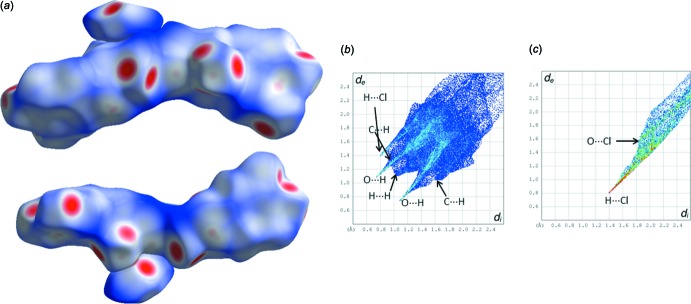
(*a*) Two different views of Hirshfeld surfaces of dimeric dopa mol­ecules along with a Cl^−^ anion, (*b*) two-dimensional fingerprint plots for complete unit of dopa and (*c*) anionic Cl^−^. Various types of contacts are indicated.

The Hirshfeld surfaces (HS) and the decomposed two-dimensional fingerprint plots have been generated, using the program *CrystalExplorer* (Wolff *et al.*, 2012 ▸), to investigate the similarities and differences in the crystal packing amongst polymorphs. The two different views of the HS diagram for the complete unit of dopa mol­ecules along with the Cl^−^ anion and the two-dimensional fingerprint plots are shown in Fig. 7 ▸.

The analysis suggests that the O⋯H contacts contribute more (41.6%) to the crystal packing when compared to other contacts with respect to the dopa mol­ecules in the title compound. The relative contributions of H⋯H, C⋯H and H⋯Cl contacts are 29, 18.6 and 6.2%, respectively, with respect to the complete unit of dopa mol­ecule. These contacts are nearly identical in the case of the bromide counterpart. The H⋯Cl and O⋯Cl contacts contributions to the Hirshfeld surface area for the Cl ion are 71.9 and 13.7%, respectively. In the bromide counterpart (III), the corres­ponding contacts are found to be 64.1 (H⋯Br) and 10.2% (O⋯Br). It is clearly seen that these contacts are lower in the bromide counterpart (III) when compared to the title salt (II).

In form (I) of the dopa HCl structure, the relative contributions of O⋯H, H⋯H, C⋯H and H⋯Cl contacts are 40.5, 25.2, 17.1 and 14.1%, respectively, with respect to the cationic dopa mol­ecule. It is worthy to note that O⋯H and H⋯H contacts are reduced by 1.1–3.8% when compared to form (II). The H⋯Cl contact is increased by 7.9% in (I) when compared to (II) of the dopa HCl structure. In (I) anionic Cl^−^, the relative contribution of H⋯Cl contacts is found be 90.4%. This is approximately 18.5 and 26% higher when compared to (II) and its bromide counterpart (III). These contacts are used to discriminate between forms (I) and (II).

## Synthesis and crystallization   

l-dopa and HCl (1:1 molar ratio) were dissolved in double-distilled water and stirred well for 6 h. The mixture was filtered and the filtrate left to evaporate slowly. Colourless block-shaped crystals of the title mol­ecular salt (II) were obtained after a growth period of 15 days.

## Refinement   

Crystal data, data collection and structure refinement details are summarized in Table 2 ▸. Since the title mol­ecular salt (I) is isotypic with its bromide counterpart (III) (Kathiravan *et al.*, 2016 ▸), it was refined with the coordinates of the dopa mol­ecule of the latter as a starting model. The Cl^−^ anion was located from a difference Fourier map. The amino and carb­oxy­lic acid H atoms were located from a difference Fourier map and freely refined. The OH groups of the dopa side chain and C-bound H atoms were treated as riding atoms and included in geometrically calculated positions: C—H = 0.93–0.98 and O—H = 0.82 Å, with *U*_iso_(H) = 1.2*U*_eq_(C) and 1.5*U*_eq_(O). The carb­oxy­lic acid O—H bond length was restrained to 0.90 (2) Å, using a DFIX option.

## Supplementary Material

Crystal structure: contains datablock(s) I. DOI: 10.1107/S2056989016016789/su5331sup1.cif

Structure factors: contains datablock(s) I. DOI: 10.1107/S2056989016016789/su5331Isup2.hkl

Supporting information file. DOI: 10.1107/S2056989016016789/su5331Isup3.cml

CCDC reference: 1511067

Additional supporting information: crystallographic information; 3D view; checkCIF report

## Figures and Tables

**Table 1:** Hydrogen-bond geometry (Å, °)

*D*—H⋯*A*	*D*—H	H⋯*A*	*D*⋯*A*	*D*—H⋯*A*
O1—H1*O*⋯O3^i^	0.82	1.98	2.746 (2)	155
O2—H2*O*⋯O1^ii^	0.82	2.33	2.999 (2)	140
O2—H2*O*⋯O2^ii^	0.82	2.16	2.8730 (8)	146
O4—H4*O*⋯O4^iii^	0.90 (3)	1.50 (3)	2.373 (2)	161 (8)
N1—H1*A*⋯Cl1^iv^	0.91 (3)	2.35 (4)	3.249 (2)	167 (3)
N1—H1*B*⋯Cl1	0.89 (3)	2.31 (3)	3.178 (2)	166 (2)
N1—H1*C*⋯O3^v^	0.90 (3)	1.93 (3)	2.7901 (19)	160 (3)

Symmetry codes: (i) 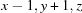; (ii) 
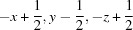
; (iii) 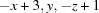; (iv) 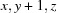; (v) 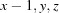.

**Table 2:** Experimental details

Crystal data	
Chemical formula	C_9_H_12_NO_4_^+^·Cl^−^·C_9_H_11_NO_4_
*M*_r_	430.83
Crystal system, space group	Monoclinic, *I*2
Temperature (K)	293
*a*, *b*, *c* (Å)	6.1768 (3), 5.4349 (3), 28.7651 (16)
β (°)	98.140 (4)
*V* (Å^3^)	955.92 (9)
*Z*	2
Radiation type	Mo *K*α
μ (mm^−1^)	0.25
Crystal size (mm)	0.30 × 0.25 × 0.20

Data collection	
Diffractometer	Bruker Kappa APEXII CCD
Absorption correction	Multi-scan (*SADABS*; Bruker, 2004 ▸)
*T*_min_, *T*_max_	0.927, 0.959
No. of measured, independent and observed [*I* > 2σ(*I*)] reflections	14303, 2982, 2623
*R*_int_	0.024
(sin θ/λ)_max_ (Å^−1^)	0.777

Refinement	
*R*[*F* ^2^ > 2σ(*F* ^2^)], *wR*(*F* ^2^), *S*	0.035, 0.094, 1.05
No. of reflections	2982
No. of parameters	151
No. of restraints	2
H-atom treatment	H atoms treated by a mixture of independent and constrained refinement
Δρ_max_, Δρ_min_ (e Å^−3^)	0.57, −0.21
Absolute structure	Flack *x* determined using 1021 quotients [(*I* ^+^) − (*I* ^−^)]/[(*I* ^+^) + (*I* ^−^)] (Parsons *et al.*, 2013 ▸)
Absolute structure parameter	0.033 (17)

Computer programs: *APEX2*, *SAINT* and *XPREP* (Bruker, 2004 ▸), *Mercury* (Macrae *et al.*, 2006 ▸), *SHELXL2014/7* (Sheldrick, 2015 ▸) and *publCIF* (Westrip, 2010 ▸). Structure solution – isomorphous replacement.

## supplementary crystallographic information

### Crystal data

**Table:**

C_9_H_12_NO_4_^+^·Cl^−^·C_9_H_11_NO_4_	*F*(000) = 452
*M**_r_* = 430.83	*D*_x_ = 1.497 Mg m^−^^3^
Monoclinic, *I*2	Mo *K*α radiation, λ = 0.71073 Å
*a* = 6.1768 (3) Å	Cell parameters from 7161 reflections
*b* = 5.4349 (3) Å	θ = 3.8–31.1°
*c* = 28.7651 (16) Å	µ = 0.25 mm^−^^1^
β = 98.140 (4)°	*T* = 293 K
*V* = 955.92 (9) Å^3^	Block, colourless
*Z* = 2	0.30 × 0.25 × 0.20 mm

### Data collection

**Table:**

Bruker Kappa APEXII CCD diffractometer	2623 reflections with *I* > 2σ(*I*)
Radiation source: fine-focus sealed tube	*R*_int_ = 0.024
ω and φ scan	θ_max_ = 33.5°, θ_min_ = 2.9°
Absorption correction: multi-scan (SADABS; Bruker, 2004)	*h* = −8→9
*T*_min_ = 0.927, *T*_max_ = 0.959	*k* = −7→7
14303 measured reflections	*l* = −39→40
2982 independent reflections	

### Refinement

**Table:**

Refinement on *F*^2^	2 restraints
Least-squares matrix: full	Hydrogen site location: mixed
*R*[*F*^2^ > 2σ(*F*^2^)] = 0.035	H atoms treated by a mixture of independent and constrained refinement
*wR*(*F*^2^) = 0.094	*w* = 1/[σ^2^(*F*_o_^2^) + (0.0541*P*)^2^ + 0.236*P*] where *P* = (*F*_o_^2^ + 2*F*_c_^2^)/3
*S* = 1.05	(Δ/σ)_max_ < 0.001
2982 reflections	Δρ_max_ = 0.57 e Å^−^^3^
151 parameters	Δρ_min_ = −0.21 e Å^−^^3^

### Special details

**Table:**

Geometry. All esds (except the esd in the dihedral angle between two l.s. planes) are estimated using the full covariance matrix. The cell esds are taken into account individually in the estimation of esds in distances, angles and torsion angles; correlations between esds in cell parameters are only used when they are defined by crystal symmetry. An approximate (isotropic) treatment of cell esds is used for estimating esds involving l.s. planes.
Refinement. Refined as a two-component inversion twin

### Fractional atomic coordinates and isotropic or equivalent isotropic displacement parameters (Å^2^)

**Table:**

	*x*	*y*	*z*	*U*_iso_*/*U*_eq_	Occ. (<1)
O1	0.3951 (2)	1.2691 (3)	0.33134 (6)	0.0290 (3)	
H1O	0.4509	1.3678	0.3510	0.044*	
O2	0.3026 (2)	0.9556 (3)	0.26334 (5)	0.0299 (3)	
H2O	0.2767	0.8411	0.2448	0.045*	
O3	1.4771 (2)	0.5509 (3)	0.41112 (5)	0.0329 (4)	
O4	1.32315 (19)	0.6323 (4)	0.47766 (4)	0.0276 (3)	
H4O	1.467 (5)	0.623 (16)	0.489 (3)	0.07 (2)*	0.5
N1	0.9230 (2)	0.6219 (4)	0.44032 (5)	0.0192 (3)	
H1A	0.937 (4)	0.751 (7)	0.4608 (11)	0.038 (8)*	
H1B	0.933 (3)	0.498 (5)	0.4608 (8)	0.013 (5)*	
H1C	0.784 (5)	0.629 (7)	0.4261 (10)	0.043 (7)*	
C1	0.8763 (3)	0.8689 (4)	0.34670 (6)	0.0203 (4)	
C2	0.7324 (3)	1.0590 (4)	0.35439 (7)	0.0216 (4)	
H2	0.7691	1.1672	0.3793	0.026*	
C3	0.5418 (3)	1.0860 (3)	0.32596 (6)	0.0202 (4)	
C4	0.4932 (3)	0.9175 (4)	0.29009 (6)	0.0207 (4)	
C5	0.6340 (3)	0.7289 (4)	0.28237 (7)	0.0237 (4)	
H5	0.5961	0.6194	0.2577	0.028*	
C6	0.8254 (3)	0.7042 (4)	0.31046 (7)	0.0238 (4)	
H6	0.9217	0.5784	0.3056	0.029*	
C7	1.0881 (3)	0.8448 (4)	0.37635 (7)	0.0231 (4)	
H7A	1.1137	0.9888	0.3963	0.028*	
H7B	1.2030	0.8373	0.3566	0.028*	
C8	1.0974 (2)	0.6136 (4)	0.40704 (6)	0.0170 (3)	
H8	1.0707	0.4692	0.3866	0.020*	
C9	1.3196 (3)	0.5949 (4)	0.43384 (6)	0.0193 (4)	
Cl1	1.0000	0.12970 (14)	0.5000	0.0428 (2)	

### Atomic displacement parameters (Å^2^)

**Table:**

	*U*^11^	*U*^22^	*U*^33^	*U*^12^	*U*^13^	*U*^23^
O1	0.0242 (7)	0.0254 (7)	0.0347 (8)	0.0052 (6)	−0.0053 (6)	−0.0063 (6)
O2	0.0210 (6)	0.0325 (8)	0.0314 (8)	0.0000 (6)	−0.0127 (5)	−0.0043 (6)
O3	0.0148 (5)	0.0560 (11)	0.0263 (7)	0.0060 (6)	−0.0025 (5)	−0.0092 (7)
O4	0.0137 (5)	0.0486 (9)	0.0186 (6)	0.0019 (7)	−0.0049 (4)	−0.0034 (7)
N1	0.0122 (6)	0.0258 (8)	0.0185 (7)	−0.0008 (6)	−0.0015 (5)	0.0018 (8)
C1	0.0176 (7)	0.0246 (9)	0.0175 (8)	−0.0016 (7)	−0.0022 (6)	0.0054 (7)
C2	0.0201 (7)	0.0237 (9)	0.0192 (8)	−0.0031 (7)	−0.0028 (6)	−0.0007 (7)
C3	0.0180 (7)	0.0205 (10)	0.0210 (8)	−0.0009 (7)	−0.0007 (6)	0.0025 (7)
C4	0.0178 (7)	0.0240 (9)	0.0188 (8)	−0.0028 (7)	−0.0029 (6)	0.0025 (7)
C5	0.0240 (9)	0.0258 (10)	0.0197 (9)	−0.0012 (8)	−0.0021 (7)	−0.0029 (7)
C6	0.0216 (8)	0.0265 (10)	0.0224 (9)	0.0041 (7)	0.0003 (7)	0.0007 (7)
C7	0.0151 (7)	0.0291 (10)	0.0233 (9)	−0.0042 (7)	−0.0038 (6)	0.0065 (8)
C8	0.0122 (6)	0.0215 (8)	0.0161 (7)	−0.0002 (7)	−0.0022 (5)	−0.0005 (7)
C9	0.0130 (6)	0.0237 (10)	0.0197 (8)	0.0003 (7)	−0.0033 (5)	−0.0012 (7)
Cl1	0.0677 (5)	0.0189 (3)	0.0390 (4)	0.000	−0.0016 (4)	0.000

### Geometric parameters (Å, º)

**Table:**

O1—C3	1.369 (2)	C1—C7	1.463 (2)
O1—H1O	0.8200	C2—C3	1.343 (2)
O2—C4	1.329 (2)	C2—H2	0.9300
O2—H2O	0.8200	C3—C4	1.380 (3)
O3—C9	1.269 (2)	C4—C5	1.382 (3)
O4—C9	1.274 (2)	C5—C6	1.341 (3)
O4—H4O	0.90 (3)	C5—H5	0.9300
N1—C8	1.540 (2)	C6—H6	0.9300
N1—H1A	0.91 (3)	C7—C8	1.532 (3)
N1—H1B	0.89 (3)	C7—H7A	0.9700
N1—H1C	0.90 (3)	C7—H7B	0.9700
C1—C6	1.376 (3)	C8—C9	1.479 (2)
C1—C2	1.401 (3)	C8—H8	0.9800
			
C3—O1—H1O	109.5	C6—C5—C4	119.90 (18)
C4—O2—H2O	109.5	C6—C5—H5	120.0
C9—O4—H4O	103 (5)	C4—C5—H5	120.0
C8—N1—H1A	114.6 (18)	C5—C6—C1	118.60 (18)
C8—N1—H1B	113.7 (14)	C5—C6—H6	120.7
H1A—N1—H1B	99 (2)	C1—C6—H6	120.7
C8—N1—H1C	115.1 (18)	C1—C7—C8	111.52 (15)
H1A—N1—H1C	105 (3)	C1—C7—H7A	109.3
H1B—N1—H1C	108 (3)	C8—C7—H7A	109.3
C6—C1—C2	121.15 (15)	C1—C7—H7B	109.3
C6—C1—C7	118.24 (18)	C8—C7—H7B	109.3
C2—C1—C7	120.58 (17)	H7A—C7—H7B	108.0
C3—C2—C1	120.37 (17)	C9—C8—C7	108.25 (15)
C3—C2—H2	119.8	C9—C8—N1	110.92 (13)
C1—C2—H2	119.8	C7—C8—N1	111.19 (16)
C2—C3—O1	123.21 (17)	C9—C8—H8	108.8
C2—C3—C4	117.50 (17)	C7—C8—H8	108.8
O1—C3—C4	119.29 (15)	N1—C8—H8	108.8
O2—C4—C3	114.24 (17)	O3—C9—O4	129.22 (14)
O2—C4—C5	123.27 (17)	O3—C9—C8	117.83 (15)
C3—C4—C5	122.47 (16)	O4—C9—C8	112.93 (14)
			
C6—C1—C2—C3	−0.8 (3)	C2—C1—C6—C5	0.0 (3)
C7—C1—C2—C3	177.45 (18)	C7—C1—C6—C5	−178.29 (19)
C1—C2—C3—O1	−179.42 (18)	C6—C1—C7—C8	−69.7 (2)
C1—C2—C3—C4	1.3 (3)	C2—C1—C7—C8	112.0 (2)
C2—C3—C4—O2	−179.88 (17)	C1—C7—C8—C9	176.83 (16)
O1—C3—C4—O2	0.8 (3)	C1—C7—C8—N1	−61.1 (2)
C2—C3—C4—C5	−1.1 (3)	C7—C8—C9—O3	−67.7 (2)
O1—C3—C4—C5	179.57 (18)	N1—C8—C9—O3	170.10 (19)
O2—C4—C5—C6	179.00 (19)	C7—C8—C9—O4	110.8 (2)
C3—C4—C5—C6	0.4 (3)	N1—C8—C9—O4	−11.4 (3)
C4—C5—C6—C1	0.2 (3)		

### Hydrogen-bond geometry (Å, º)

**Table:**

*D*—H···*A*	*D*—H	H···*A*	*D*···*A*	*D*—H···*A*
O1—H1*O*···O3^i^	0.82	1.98	2.746 (2)	155
O2—H2*O*···O1^ii^	0.82	2.33	2.999 (2)	140
O2—H2*O*···O2^ii^	0.82	2.16	2.8730 (8)	146
O4—H4*O*···O4^iii^	0.90 (3)	1.50 (3)	2.373 (2)	161 (8)
N1—H1*A*···Cl1^iv^	0.91 (3)	2.35 (4)	3.249 (2)	167 (3)
N1—H1*B*···Cl1	0.89 (3)	2.31 (3)	3.178 (2)	166 (2)
N1—H1*C*···O3^v^	0.90 (3)	1.93 (3)	2.7901 (19)	160 (3)

Symmetry codes: (i) *x*−1, *y*+1, *z*; (ii) −*x*+1/2, *y*−1/2, −*z*+1/2; (iii) −*x*+3, *y*, −*z*+1; (iv) *x*, *y*+1, *z*; (v) *x*−1, *y*, *z*.
